# Usefulness of Novel Atherogenic Lipid Indices for the Evaluation of Metabolic Status Leading to Coronary Heart Disease in a Real-World Survey of the Japanese Population

**DOI:** 10.3390/healthcare10040747

**Published:** 2022-04-17

**Authors:** Isamu Matsunaga, Miyuki Ando, Yuki Tsubakimoto, Miyuki Nagasawa, Yoshimasa Kurumi

**Affiliations:** Healthcare Centre, JCHO Shiga Hospital, Fujimidai 16-1, Otsu 520-0846, Shiga, Japan

**Keywords:** atherogenesis, metabolic status, lipid index, coronary heart disease, medical check-up

## Abstract

We evaluated the usefulness of two novel cholesterol-triglyceride subgroup (CTS) indices, CTS_qlt_ and CTS_qnt_, that potentially reflect the metabolic status regarding risk of coronary heart disease (CHD) using a retrospective longitudinal study of the Japanese general population. We recruited 12,373 individuals from the annual users of our healthcare center. Among them, the first onset of CHD was recorded in 131 individuals between April 2014 and March 2020. The multivariate Cox proportional hazards regression analyses for all normalized lipid indices revealed that the CTS_qnt_ index showed a comparable hazard ratio for the CHD outcome to non-high-density lipoprotein cholesterol (nonHDL-c) and triglycerides. The HR of the CTS_qlt_ index was significantly lower than for CTS_qnt_, but still comparable to that for low-density lipoprotein cholesterol (LDL-c). In comparison with the other indices, CTS_qlt_ is more sensitive to risk increment while the index value increases. Linear regression analyses for the CTS indices and previously known lipid indices suggest that the CTS_qnt_ and CTS_qlt_ indices reflect the quantity of atherogenic lipoproteins and particle size (quality) of smaller and denser LDLs, respectively. Furthermore, the CTS_qnt_/HDL-c index can be used as a comprehensive risk indicator that may represent the status of lipid metabolism determined by the CTS_qlt_ and CTS_qnt_ indices and thus may be useful for screening. The CTS indices can be used to evaluate the metabolic status of individuals, which may increase the risk of future CHD.

## 1. Introduction

Low-density lipoprotein (LDL) delivers cholesterol and other lipids from the liver, the primary production site of a precursor of LDL, very low-density lipoprotein (VLDL), to the periphery. In contrast, high-density lipoprotein (HDL) transports cholesterol from the periphery to the liver. This transport of lipoproteins between the liver and periphery maintains lipid homeostasis [1,2]. Lipoproteins with other densities, apoprotein types, and lipid composition do not disturb the lipid homeostasis in the physiological condition. However, excess amounts of normal or aberrant lipoproteins disturb the homeostasis, leading to abnormal lipid deposition in the periphery and liver. Cholesterol deposition in the arterial wall is one of the typical features of atherosclerosis. Although atherosclerosis generates through complex mechanisms [3,4], it is obvious that excess amount of LDL-cholesterol (LDL-c) is one of the causes of atherosclerosis and subsequent coronary heart disease (CHD). Statins improves the CHD outcome by decreasing the LDL-c levels. However, the LDL-c levels are only partially reduced and up to 40% of statin-treated patients still develop CHD [5,6]. Furthermore, many CHD patients may not have significantly increased LDL-c levels. Therefore, researchers have investigated other factors that may explain the residual risk of CHD caused by atherosclerosis. Recent studies have focused on blood triglyceride levels, which are derived from TG-rich lipoproteins (TRLs), including VLDL and chylomicrons [6,7,8]. VLDL and chylomicron macromolecules, however, are too big to pass through the arterial endothelium from the blood stream to enter into the arterial wall. Thus, they are not cholesterol sources in the physiological condition, but metabolic disturbances produce smaller TRL molecules, called remnant lipoproteins, that can cross the arterial wall. Moreover, persistence of high blood TG level makes the LDL molecules smaller and denser by the activity of lipases to catabolize TGs. The small dense LDL (sdLDL) thereby produced is easily oxidized to generate the highly harmful aberrant lipoprotein, oxidized LDL [9]. Because both remnant lipoproteins and sdLDL are highly atherogenic, their levels are clinically important to prevent or manage atherosclerotic diseases. However, the methods of laboratory measurement of remnant-cholesterol (remnant-c) and sdLDL-cholesterol (sdLDL-c) levels are not standardized as the routine laboratory tests. Therefore, alternative markers are required to estimate cholesterol levels in the atherogenic lipoproteins.

We engaged in medical check-up programs, including occupational healthcare programs for workers and personal healthcare programs for the general population in Shiga prefecture, Japan, and provided useful information regarding healthcare management and disease prevention. Since cardiovascular diseases provoked by atherosclerosis are leading causes of death and disability, and lead to personal suffering and socioeconomic loss, we focused on encouraging individuals without CHD but with risk factors for CHD to modify their lifestyle to prevent CHD and stroke through our check-up service. Lipid biomarkers are useful, but the ideal biomarker should be easily measured by routine laboratory tests at a low cost. For this purpose, we measured LDL-c, HDL-c, TG, and nonHDL-c levels, in addition to encouragement of smoking cessation, control of blood pressure and blood glucose levels with suitable exercise and diet, and advice to eliminate other risk factors. However, the prediction of future risk using the traditional lipid biomarkers is not always accurate because the biomarkers are components of biomolecules that are in a dynamic balance and vary between individuals. Additionally, these biomarkers are essential for the homeostasis in the physiological condition and are not only biomarkers for atherosclerosis. We aimed to identify biomarkers that reflect lipid metabolism in the pathological condition and can be easily measured. While providing healthcare information to individuals using our service, we identified some patterns on the scatter plot of TG against LDL-c for more than 10,000 individuals in a year. The distribution appeared to be separated into subgroups characterized by two indices calculated with simple formulas and designated as the cholesterol-triglyceride-subgroup (CTS) indices. Herein, we investigated the usefulness of CTS indices for predicting CHD outcome by comparing them with previously known lipid indices in a retrospective longitudinal study, and explored the basis of these indices in lipid metabolism. The aim of this study was to evaluate the usefulness of novel atherogenic indices that potentially reflect the metabolic status of apparently healthy people that increase the risk of atherosclerotic disorders, including CHD. For a more precise comparison among the lipid indices, including traditional lipid parameters (such as TC, LDL-c, HDL-c, and TG), we performed the Box–Cox transformation for each lipid index, to convert skewed distribution into a normal distribution. Then, we compared the HRs per one standard deviation (1 SD) of the indices. 

## 2. Materials and Methods

### 2.1. Study Design

This observational study included participants who underwent an annual health checkup at our facility between April 2013 and March 2020. Most participants were residents of the Shiga prefecture or neighboring cities, Japan. The participants in this study were recruited from annual users of our service from April 2019 to March 2020. Then, we retrospectively excluded the participants who already had CHD history in 2013 or who did not have complete baseline data in 2013 as described below. We identified individuals with a history of CHD (disease group; 406 individuals) and without a history of CHD (control group; 15,546 individuals) who used our service between April 2019 and March 2020. The CHD history was checked annually during an interview or through medical records using a questionnaire that collected information on age at onset, current medication use for CHD, and general items (e.g., age, sex, and current or past smoking). We excluded individuals with CHD history or without complete baseline data (including age, sex, smoking status, systolic and diastolic blood pressures, blood glucose levels (fasting blood glucose level and/or HbA1c), and TG, total cholesterol (TC), LDL-c, and HDL-c levels) collected in 2013. In total, 131 individuals were included in the disease group, with CHD onset reported in 2014–2020, and 12,242 individuals in the control group. In this study, we estimated the ability of lipid indices to predict the first onset of CHD that did not prevent patients continuing to use our services. 

### 2.2. Measurements

Lipid, blood glucose, and HbA1c levels were measured by routine laboratory tests. LDL-c was measured by the selective solubilization method using MetaboLead LDL-c (Kyowa Medix, Tokyo, Japan). Non-HDL-c was calculated as TC minus HDL-c. Atherogenic lipoprotein cholesterols were calculated as calculated sdLDL-c plus nonLDL-nonHDL cholesterol. SdLDL-c was calculated as previously reported [10]. NonLDL-nonHDL cholesterol was calculated as TC minus LDL-c minus HDL-c. 

Two CTS indices, designated CTS_qlt_ and CTS_qnt_, were calculated as follows:

CTS_qlt_ index = TG^2^/(LDL-c × 100)

CTS_qnt_ index = 0.2 × LDL-c + 0.15 × TG

Of the 12,373 study participants, blood specimens were collected after more than 10 h of fasting from 10,297 participants (109 from the disease group) and residual 2076 specimens were collected within 10 h after the last meal. Blood glucose and HbA1c levels were categorized into lower (L), middle (M), and higher (H) categories (Table S1); the higher category was used for the regression analyses. Systolic and diastolic blood pressures were categorized into lower (L), middle (M), and higher (H) categories (Table S1); the higher category was used for regression analyses. 

### 2.3. Statistical Analyses

We intended to compare hazard ratios (HRs) per 1 SD of the lipid indices for the CHD outcome, because one unit in the whole range was significantly different between the lipid indices. However, the distribution of some lipid indices (e.g., CTS_qlt_) was extremely skewed and was therefore not suitable for use to calculate the SD to evaluate the HRs. Therefore, we used the Box–Cox transformation method to transform the distribution of the lipid index close to normal distribution [11]. The Box–Cox transformation was as follows:

X = (x^λ^ − 1)/λ (λ ≠ 0)

X = ln(x) (λ = 0)

where x is the original value of the lipid index before the transformation. We determined the appropriate λ value for each index using *p* values from the Kolmogorov–Smirnov test and a Q-Q plot analysis (Figure S1 for CTS_qlt_ index). Using the transformed lipid indices, we performed Cox proportional hazards regression analyses to obtain the HRs per 1 SD. In the multivariate regression analyses, age, sex, smoking history, and categories of blood pressure and blood glucose levels were adjusted. Difference in the HR values for the two normalized indices was evaluated using the Welch’s test. 

For the latter analyses, we divided the population in this study into three group (groups 1, 2, and 3 from lower to higher) divided by 33.3 percentile and 66.6 percentile for each lipid index that is not transformed. Then, we estimated the risk increment compared to group 1. For other analyses, Chi-square test and Mann–Whitney U test were used for the categorical variables and continuous variables, respectively. All analyses were performed using the EZR software (Saitama Medical Centre, Jichi Medical University, Saitama, Japan), which is a modified version of R commander, designed to add statistical functions for biostatistics [12].

## 3. Results

### 3.1. Comparison of HRs for the Lipid Indices

Table 1 shows the summary of the study population. Age, sex, smoking history, and categories of blood pressure and blood glucose levels significantly differed between the disease group and control group. To precisely estimate the HRs of the lipid indices, we adjusted them in the multivariate regression analyses. The HR for CTS_qnt_ index was not significantly different from those of the TG and nonHDL-c in the multivariate Cox proportional hazards regression analyses (Table 2). The HR for the CTS_qlt_ index, which was lower than that for CTS_qnt_ (*p* < 0.05), was comparable to that of the traditional atherogenic index, LDL-c. The HR for TC was the lowest among the indices evaluated, but the 95% confidence interval (CI) was greater than 1, indicating that the increase in TC levels increased the CHD risk. In contrast, the 95% CI for HDL-c was lower than 1, so the increase in HDL-c levels decreased the CHD risk, similar to previous studies [2]. We observed little effects of medications for HRs of the lipid indices in our model.

The combination of a lipid parameter with HDL-c, in which a parameter is divided by HDL-c, enhances the predictive ability of the index by increasing the HR for CHD. Both the LDL-c/HDL-c and TG/HDL-c indices have been proposed as good indicators of atherogenicity [13,14,15,16]. Therefore, the LDL-c/HDL-c and TG/HDL-c indices are likely to be better predictors of CHD, as evidenced by their HRs (Table 3). In this study, we found that the combination of CTS_qnt_ and HDL-c showed comparable ability with LDL-c/HDL-c and TG/HDL-c to predict the CHD outcome. There was no statistically significant difference among HRs of these three indices.

To estimate relative risk in a lipid parameter with skewed distribution, we divided the target population into quartiles (i.e., G-1, G-2, and G-3; divided by 33.3 percentile and 66.6 percentile for CTS_qlt_, TG/HDL-c, CTS_qnt_, and nonHDL-c) (Table 4). The HRs in the higher group (G-3) and lower group (G-1) were similar in all indices, but the HR in the middle group (G-2) of the CTS_qlt_ index was considerably higher. Notably, the magnitude of change in the HR between G-1 and G-2 and between G-1 and G-3 is more important in each index rather than their absolute values. This result was confirmed by graphical analyses using Kaplan–Meier curves (Figure S2). In addition, we confirmed the proportionality of hazards using a log–log plot. These results suggested that the CTS_qlt_ index was more sensitive for predicting the CHD risk, especially for the middle group.

### 3.2. Characteristics of CTS Indices

**Figure 1:**
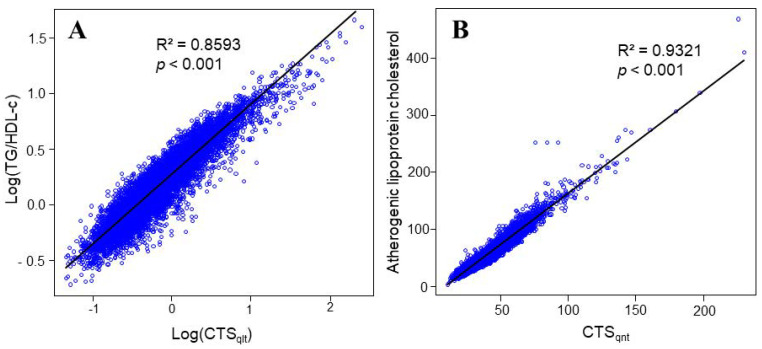
Linear regression analyses of CTSqlt to TG/HDL-c as a response variable in common logarithmic forms (**A**) and CTSqnt to the atherogenic lipoprotein cholesterol (**B**). Regression lines (black lines) were determined using the least squares method.

To understand the possible biological basis of the CTS_qlt_ and CTS_qnt_ indices, we investigated their relationships with previously known lipid indices. The CTS_qlt_ index showed the best correlation with TG/HDL-c in the logarithmically transformed forms, in which the coefficient of determination was 0.8593 for log(TG/HDL-c) as a response variable (Figure 1A). The log(TG/HDL-c) has been designated as an “atherogenic index in plasma (AIP)” and a good predictor for CHD [17]. More importantly, AIP showed very good correlation with the LDL particle size [18], with larger AIP values suggesting the production of potentially atherogenic smaller and denser LDLs. For the CTS_qnt_ index, we considered a combination of calculated sdLDL-c and nonLDL-nonHDL cholesterol as a response variable, which is designated as an “atherogenic lipoprotein cholesterol” in this study. Surprisingly, the CTS_qnt_ index and atherogenic lipoprotein cholesterol showed an extremely high correlation coefficient (Figure 1B). Thus, the CTS_qnt_ index appears to be another marker of the atherogenic lipoprotein cholesterol, but it should be noted that this index does not indicate the quantity of the lipoprotein cholesterol. In addition, we found that sdLDL-c can be estimated using the following formula: 0.2 LDL-c + 0.1 TG (Pearson’s correlation coefficient = 0.9863; 95% CI = 0.9961–0.9964; *p* < 0.001). Therefore, nonLDL-nonHDL-c is expressed by the residual 0.05 TG (Pearson’s correlation coefficient = 0.876; 95% CI = 0.872–0.880; *p* < 0.001).

**Figure 2:**
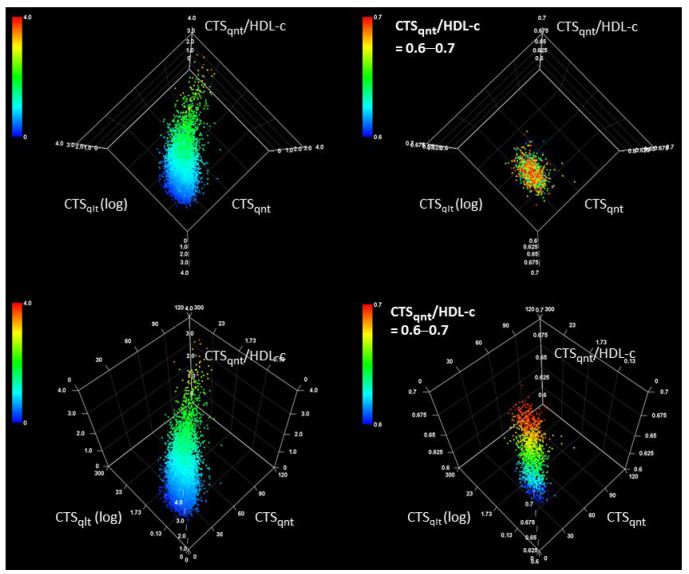
Three-dimensional scattered plots for the three CTS indices. The two images on the (**left**) display the same graph as it would appear from different directions. Individuals with higher CTSqnt/HDL-c are shown as red-colored dots. The color of dots gradually changed to blue as the CTSqnt/HDL-c values decreased. The CTSqlt axis is common logarithmically scaled. The (**right**) two graphs are scattered plots when the CTSqnt/HDL-c value is limited to 0.6–0.7. Individuals with CTSqnt/HDL-c of 0.7 are shown in red color, and the color gradually changes to blue for CTSqnt/HDL-c value of 0.6.

To understand the correlations between CTS_qlt_, CTS_qnt_, and CTS_qnt_/HDL-c, we observed the relationship among these indices using a three-dimensional graphical analysis (Figure 2), in which an axis of the CTS_qlt_ index is logarithmically expressed. The Pearson’s correlation coefficients for the relationships are as follows: 0.780 (95% CI = 0.773–0.787; *p* < 0.001) for CTS_qnt_ and log(CTS_qlt_); 0.904 (95% CI = 0.901–0.907; *p* < 0.001) for CTS_qnt_ and CTS_qnt_/HDL-c; and 0.763 (95% CI = 0.756–0.770; *p* < 0.001) for log(CTS_qlt_) and CTS_qnt_/HDL-c. As shown in Figure 2, the distribution was linear, not distorted. Within the limited range of the CTS_qnt_/HDL-c (e.g., 0.6–0.7 in Figure 2), the shape of the distribution appeared like a vertical column on the CTS_qlt_-CTS_qnt_ plane. This result suggests that the CTS_qnt_/HDL-c index represents the metabolic status of individuals restricted within the range of CTS_qnt_ and CTS_qlt_. In other words, the CTS_qnt_/HDL-c index provides some information regarding the metabolic status of an individual, which can be further explored by the use of CTS_qnt_ and CTS_qlt_ indices.

## 4. Discussion

The CTS index is comparable to the previously known lipid indices in terms of ability to predict the CHD outcome for the Japanese general population. In particular, the LDL-c/HDL-c and TG/HDL-c indices have been reported as good lipid predictors of atherogenicity. This study found that the CTS_qnt_/HDL-c index was comparable to the aforementioned indices. Interestingly, the formula of the CTS_qnt_/HDL-c index can be deformed as 0.2 × (LDL-c/HDL-c) + 0.15 × (TG/HDL-c), which is understandable as a consolidated index of LDL-c/HDL-c and TG/HDL-c with the addition of weights. These results indicate the CTS_qnt_/HDL-c index is as useful as the previously known atherogenic indices. 

Both CTS_qlt_ and CTS_qnt_ indices have characteristic patterns of distribution and biochemical natures. The CTS_qlt_ index may reflect lipoprotein particle size, as presumed from its close correlation with the TG/HDL-c index in their logarithmically transformed forms. The HR/1 SD for the CTS_qlt_ index was less than TG/HDL-c (Table 1 and Table 2). However, focusing on the change in the relative risk along with the increment (or decrement) of those indices, the CTS_qlt_ index appears to be more sensitive to the changes over the middle group than the TG/HDL-c index, suggesting that the CTS_qlt_ index is a better indicator (Table 4 and Figure S2). The CTS_qnt_ index is a marker of atherogenic lipoprotein cholesterol and consists of calculated sdLDL-c and nonLDL-nonHDL-c. The nonLDL-nonHDL-c is recognized as the calculated remnant-c [19,20]; therefore, the atherogenic lipoprotein cholesterol in this study is a consolidated expression of sdLDL-c and remnant-c. The most important issue is that the formula of CTS_qnt_ index includes TG. Hence, it is reasonable that both sdLDL-c and remnant-c are affected by the increased TG. Notably, both sdLDL-c and remnant-c are calculated only with cholesterol parameters. Srisawasdi et al. [10] proposed a complicated formula to estimate sdLDL-c using several cholesterol parameters by a regression analysis of the measured sdLDL-c. However, our results suggest that this formula includes a hidden TG parameter. For the calculated remnant-c, it may be assumed that this is an expression of cholesterol in VLDL and thus, it can be estimated by TG of VLDL, particularly in the fasting condition. The usefulness of both calculated sdLDL-c and remnant-c for predicting the risk of CHD has previously been reported [21,22]. It is suggested that the CTS_qnt_ index proposed in this study is a consolidated and more useful indicator for atherogenicity than the isolated use of calculated sdLDL-c or remnant-c. 

**Figure 3:**
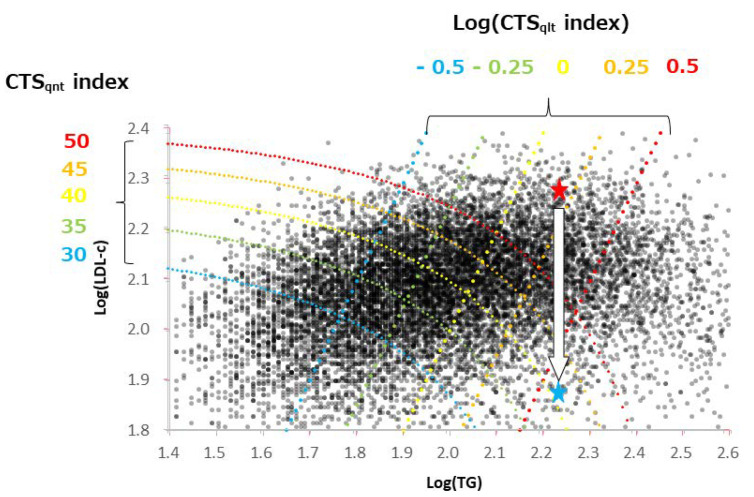
The values of CTSqlt and CTSqnt indices are illustrated as colored dotted lines on a scattered plot with log(TG) as the X axis and log(LDL-c) as the Y axis. Gray dots are the individual subjects. A subject with the high CTSqnt index is shown as a red star symbol. After treatment to reduce LDL-c but without a decrease in TG, the metabolic status of the individual is shown as a blue star symbol.

We investigated the relationships between CTS_qlt_, CTS_qnt_, and CTS_qnt_/HDL-c (Figure 2). CTS_qlt_ and CTS_qnt_ showed related metabolic conditions, but differed in their biochemical nature. The CTS_qnt_ index reflects the quantity of the atherogenic lipoproteins, whereas the CTS_qlt_ index probably reflects the size of LDL macromolecules. These two indices can be illustrated using a scattered plot with log(TG) as the X axis and log(LDL-c) as the Y axis (Figure 3), in which gray dots are the individual subjects. Considering an individual with high CTS_qnt_ index (red-colored star symbol) who is administered a treatment that reduces LDL-c without decreasing TG (blue-colored star symbol), the CTS_qnt_ index will definitely decrease, but the CTS_qlt_ index will increase. This suggests that a treatment targeting only the LDL-c without affecting blood TG levels may be less beneficial and may even be harmful. In this point of view, reduction of TG appears to be very important together with decrement of LDL-c by statin treatment. Though several pharmacological interventions including use of fibrate, omega-3 fatty acid, or niacin have been attempted as the TG-lowering therapy [7], non-pharmacological approaches including adjustment of quantity and quality of daily diets, controlled exercise to consume inner fat, and maybe nutraceuticals, such as fish oils should be encouraged for apparently healthy individuals in every health check-up service. The CTS_qnt_/HDL-c index may represent the status of lipid metabolism determined by the CTS_qlt_ and CTS_qnt_ indices in an individual. Therefore, we may use the CTS_qnt_/HDL-c index as a comprehensive indicator of the metabolic status.

Herein, we described novel atherogenic indices that are potentially useful to predict the risk of CHD. Our study revealed that three CTS indices are comparable to previously reported atherogenic indices in terms of their prediction ability for CHD. More importantly, it is suggested that these CTS indices are better indicators of the metabolic status that predisposes to atherosclerosis than the previously known indices and lipid parameters. Thus, the CTS indices may be superior to the previously known lipid indices for the evaluation of the metabolic status of individuals that may lead to CHD in the future. This information may be used to prevent CHD by advising the appropriate lifestyle changes. 

The CTS indices proposed in this study are comparable indicators of the CHD risk to previously reported atherogenic indices, but the CTS_qlt_ and CTS_qnt_ indices more directly reflect the metabolic status that predisposes to CHD than the previously known indices. The CTS_qnt_/HDL-c index can be recommended as a screening indicator. However, we need more comprehensive and controlled prospective studies involving precisely diagnosed clinical entities, including severe or fatal CHD, to confirm our results. Additionally, we need to evaluate how well the CTS indices reflect the properties of the atherogenic lipoproteins as compared to direct biochemical measurements.

## Figures and Tables

**Table 1:** Baseline data.

	Category	Disease Grozup	Control Group	*p*
Number		131	12,242	
Sex (%)	Male	104 (79.4)	8103 (66.2)	0.001
	Female	27 (20.6)	4139 (33.8)	
Smoking history (%)	No	30 (22.9)	4640 (37.9)	<0.001
	Yes	101 (77.1)	7602 (62.1)	
Blood pressure (%)	L	63 (48.1)	9343 (76.3)	<0.001
	M	43 (32.8)	2071 (16.9)	
	H	25 (19.1)	828 (6.8)	
Blood sugar (%)	L	97 (74.0)	11,093 (90.6)	<0.001
	M	12 (9.2)	640 (5.2)	
	H	22 (16.8)	509 (4.2)	
Age		56.04 [49.95, 61.98]	48.00 [40.99, 55.99]	<0.001
TC		214.00 [192.00, 235.50]	206.00 [185.00, 229.00]	0.003
TG		116.00 [84.50, 161.50]	87.00 [61.00, 131.00]	<0.001
LDL-c		129.00 [109.50, 149.50]	121.00 [101.00, 142.00]	0.002
HDL-c		54.00 [45.50, 67.50]	62.00 [51.00, 74.00]	<0.001
NonHDL-c		156.00 [135.00, 182.00]	142.00 [119.00, 167.00]	<0.001
LDL-c/HDL-c		2.29 [1.78, 3.01]	1.96 [1.48, 2.56]	<0.001
TG/HDL-c		2.19 [1.44, 3.54]	1.41 [0.86, 2.42]	<0.001
CTS_qnt_		45.80 [36.60, 55.05]	38.60 [31.40, 47.40]	<0.001
CTS_qlt_		0.99 [0.60, 1.96]	0.63 [0.32, 1.36]	<0.001
CTS_qnt_/HDL-c		0.81 [0.58, 1.15]	0.62 [0.44, 0.89]	<0.001

Chi-square test and Mann–Whitney U test were applied to the categorical and continuous variables, respectively. The median value is shown for continuous variables and the first and third quartiles are shown in the parenthesis.

**Table 2:** Cox proportional hazards regression analyses of various indices after the Box–Cox transformation for CHD outcomes.

		Univariate			Multivariate		
Index	λ *^1^	HR (/1 SD)	95% CI	*p*	HR (/1 SD)	95% CI	*p*
CTS_qnt_	−0.2	1.595	1.352–1.881	<0.001	1.354	1.131–1.622	0.001
TG	0.3	1.655	1.393–1.967	<0.001	1.350	1.114–1.636	0.002
NonHDL-c	0.4	1.512	1.277–1.790	<0.001	1.349	1.134–1.604	<0.001
CTS_qlt_	−0.2	1.587	1.338–1.893	<0.001	1.281 *^2^	1.060–1.549	0011
LDL-c	0.6	1.325	1.117–1.568	0.001	1.270	1.078–1.506	0.005
TC	0.3	1.294	1.117–1.530	0.003	1.214 *^3^	1.024–1.439	0.025
HDL-c	−0.1	0.673	0.567–0.798	<0.001	0.741	0.616–0.891	0.001

CHD, 131 cases; control, 12,242 cases during 2013–2020. The indices are normalized by the Box–Cox transformation. Note that the hazard ratios are expressed as per 1 SD. The multivariate model is adjusted by age, sex, smoking history, and categories of blood pressure and blood glucose levels. *^1^, The values in the Box–Cox transformation. *^2^, Significantly lower than CTS_qnt_ (*p* < 0.05). *^3^, significantly lower than CTS_qlt_ (*p* < 0.05).

**Table 3:** Cox proportional regression analyses of various indices after the Box–Cox transformation for CHD outcomes.

		Univariate			Multivariate		
Index	λ *^1^	HR (/1 SD)	95% CI	*p*	HR (/1 SD)	95% CI	*p*
LDL-c/HDL-c	0.3	1.571	1.323–1.866	<0.001	1.454	1.212–1.744	<0.001
CTS_qnt_/HDL-c	−0.1	1.642	1.388–1.943	<0.001	1.428	1.186–1.721	<0.001
TG/HDL-c	−0.3	1.685	1.416–2.004	<0.001	1.411	1.161–1.714	<0.001

CHD, 131 cases; control, 12,242 cases during 2013–2020. The indices are normalized by the Box-Cox transformation. Note that the hazard ratios are expressed as per 1 SD. The multivariate model is adjusted by age, sex, smoking history, and categories of blood pressure and blood glucose levels. *^1^, The values in the Box–Cox transformation.

**Table 4:** Hazard ratios of middle and higher groups versus lower group of various indices for CHD outcomes.

		Groups *^1^		
Index		G-1	G-2	G-3
CTS_qlt_	Min to Max	0.038–0.412	0.412–1.019	1.019–252.001
	HR (vs. G-1) *^2^	-	2.331	2.295
	(95% CI)	-	(1.321–4.112)	(1.299–4.056)
	*p* (vs. G-1)	-	0.003	0.004
TG/HDL-c	Min to Max	0.173–1.015	1.015–1.982	1.982–46.800
	HR (vs. G-1) *^2^	-	1.775	2.387
	(95% CI)	-	(1.016–3.101)	(1.386–4.112)
	*p* (vs. G-1)	-	0.044	0.002
CTS_qnt_	Min to Max	11.2–33.8	33.9–44.1	44.2–230.4
	HR (vs. G-1) *^2^	-	1.446	2.190
	(95% CI)	-	(0.849–2.461)	(1.332–3.601)
	*p* (vs. G-1)	-	0.175	0.002
NonHDL-c	Min to Max	39–127	128–158	159–378
	HR (vs. G-1) *^2^	-	1.451	1.828
	(95% CI)	-	(0.893–2.902)	(1.152–2.902)
	*p* (vs. G-1)	-	0.133	0.010

*^1^, The population in this study was divided by the 33.3 and 66.6 percentiles for each index to create three groups. Group 1 (G-1) and group 2 (G-2) include the 33.3 percentile and 66.6 percentile values, respectively. *^2^, Hazard ratios (HRs) are calculated by Cox proportional hazards regression analyses. The Cox proportional model is adjusted by age, sex, smoking history, and categories of blood pressure and blood glucose levels.

## Data Availability

The data presented in this study are available from the corresponding author on reasonable request. The data are not publicly available due to the restriction from the Institutional Review Board of JCHO Shiga Hospital.

## Supplementary Materials

The following supporting information can be downloaded at: https://www.mdpi.com/article/10.3390/healthcare10040747/s1, Figure S1: The Q-Q plots for the CTS_qlt_ index before (A) and after (B) the Box-Cox transformation. The λ value is –0.2; Figure S2: Kaplan-Meier curves of cumulative CHD incidence in the groups of CTS_qlt_ (A), TG/HDL-c (B), CTS_qnt_ (C), and nonHDL-c (D). The study population is divided by 33.3 and 66.6 percentiles of each index. Groups 1, 2, and 3 are shown as blue, green, and red lines, respectively; Table S1: Categories of blood pressure and blood glucose levels.
