# Supplementary material for: Usefulness of Novel Atherogenic Lipid Indices for the Evaluation of Metabolic Status Leading to Coronary Heart Disease in a Real-World Survey of the Japanese Population
Source: Healthcare (Basel). 2022 Apr 17;10(4):747. doi: 10.3390/healthcare10040747 (PMC9029713; doi:10.3390/healthcare10040747)
Supplement: Supplementary file 1 [file healthcare-10-00747-s001.zip › healthcare-1613836-supplementary.pdf]

healthcare

MDPI

Supplementary Materials

Figure S1

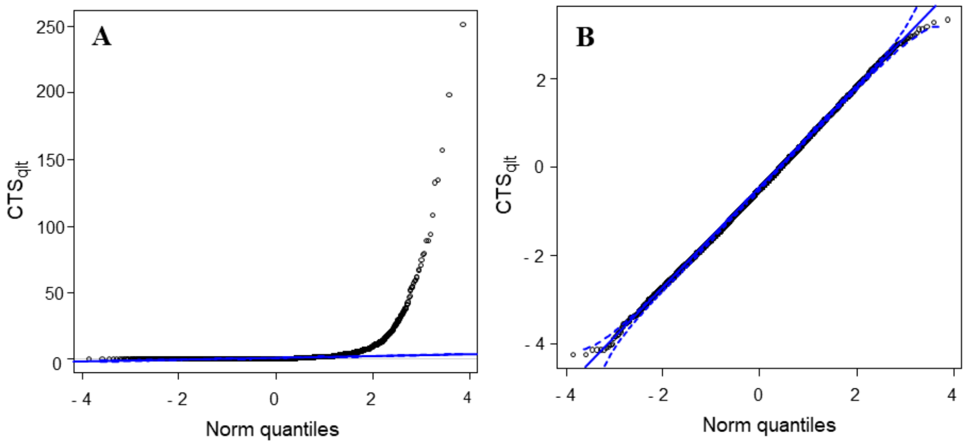

Figure S1. The Q-Q plots for the CTS_{qlt} index before (A) and after (B) the Box-Cox transformation. The λ value is –0.2.

Figure S2

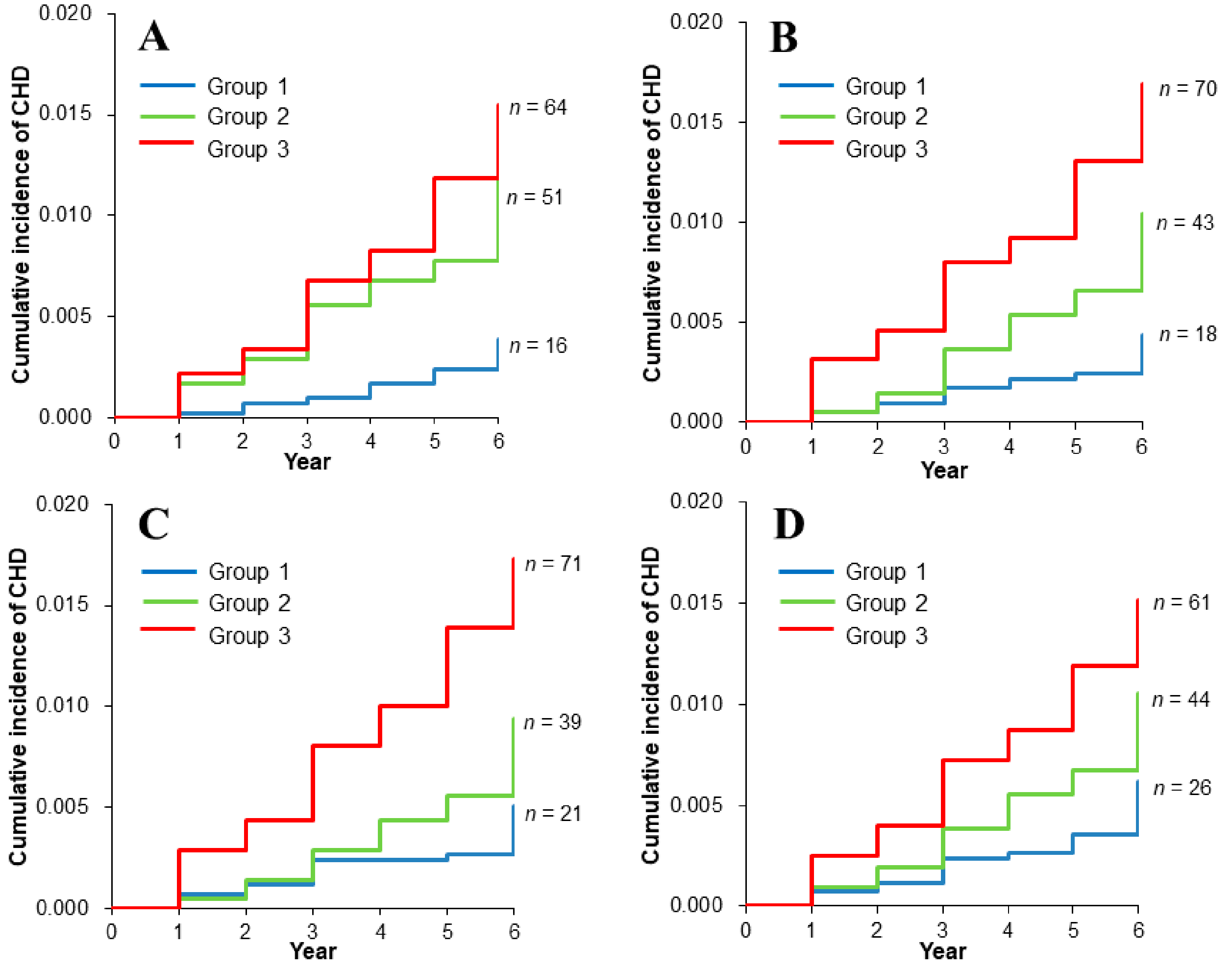

Figure S2. Kaplan-Meier curves of cumulative CHD incidence in the groups of CTS_{qlt} (A), TG/HDL-c (B), CTS_{qnt} (C), and nonHDL-c (D). The study population is divided by 33.3 and 66.6 percentiles of each index. Groups 1, 2, and 3 are shown as blue, green, and red lines, respectively. .

Table S1. Categories of blood pressure and blood glucose levels.

Factors	Variables	Category		
		L	M	H
Blood pressure[*1]	Systolic (mmHg)	–139	140–159	160–
	Diastolic (mmHg)	–89	90–99	100–
Blood sugar (BS) [*2]	Fasting BS (mg/dL) [*3]	–109	110–125	126–
	HbA1c (%)	–6.0	6.1–6.4	6.5–

*1, Higher category of systolic or diastolic blood pressure in each subject is chosen for regression analyses.

*2, Higher category of fasting blood glucose or HbA1c level is chosen in each subject for regression analyses.

*3, Fasting for more than 10 hours.
